# Supplementary figures and images for: Tissue memory CD4+ T cells expressing IL-7 receptor-alpha (CD127) preferentially support latent HIV-1 infection
Source: PLoS Pathog. 2020 Apr 30;16(4):e1008450. doi: 10.1371/journal.ppat.1008450 (PMC7192375; doi:10.1371/journal.ppat.1008450)

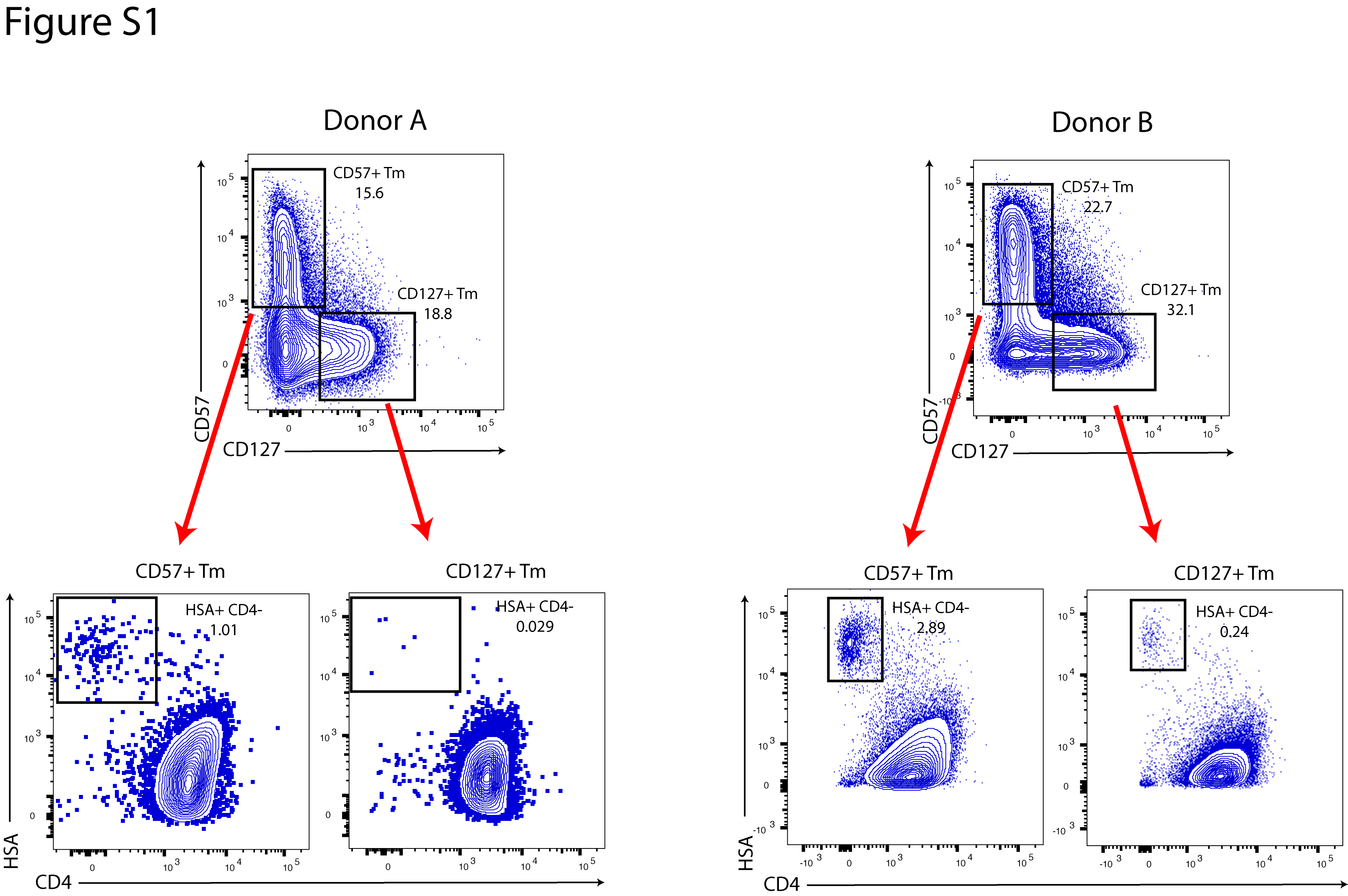

Supplement: S1 Fig — HLACs were exposed for 3 days to the CCR5-tropic reporter virus F4.HSA, after which the CD57+ Tm and CD127+ Tm populations were assessed for infection levels. Shown are results from two independent donors. Note that despite the higher frequencies of CD127+ Tm cells relative to CD57+ Tm cells, the infection rates were markedly lower in the former. (TIF) [file ppat.1008450.s001.tif]

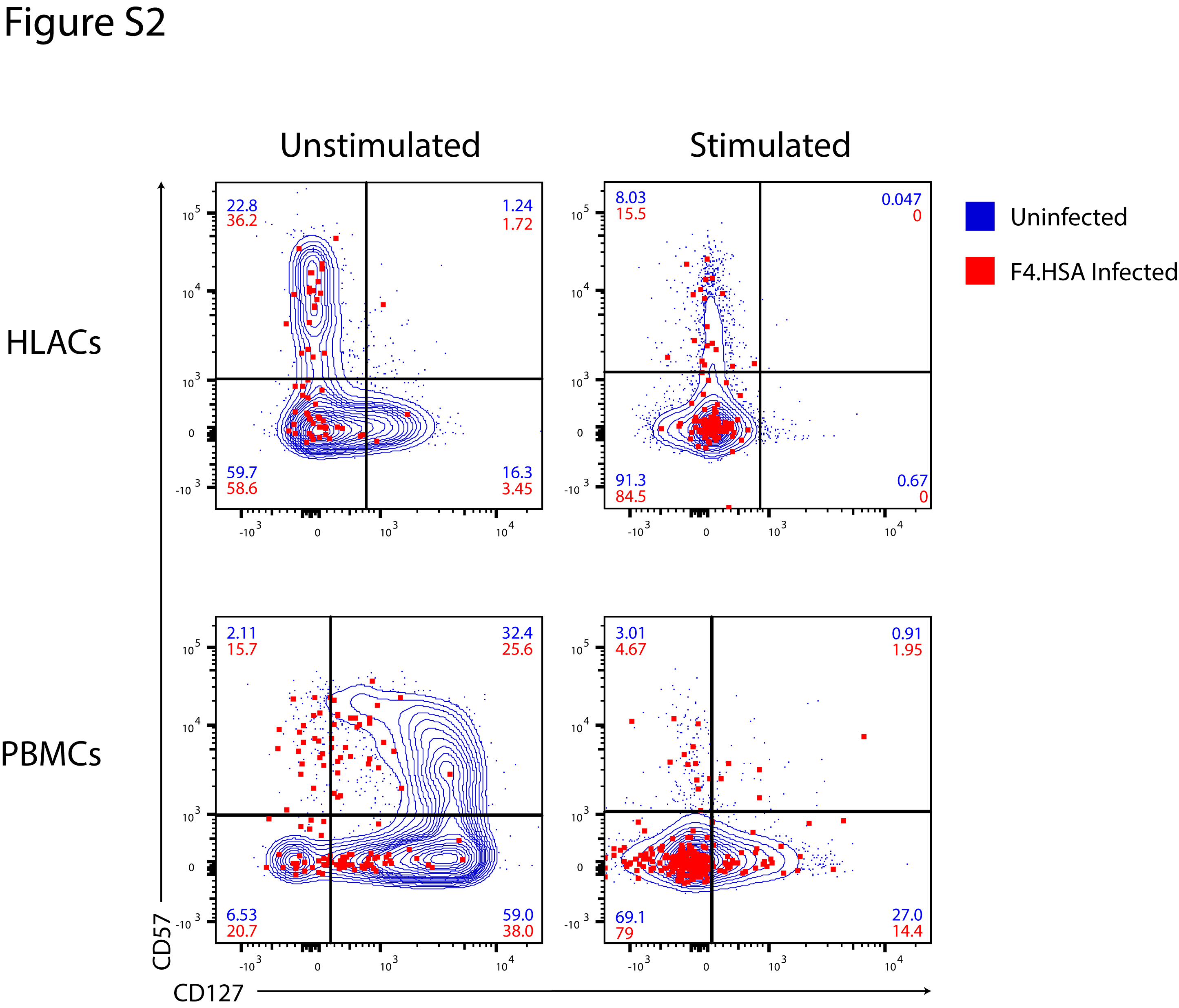

Supplement: S2 Fig — Unstimulated or PHA-stimulated HLACs or PBMCs were mock-treated or infected for 3 days with F4.HSA. Expression levels of CD57 and CD127 were then compared in uninfected memory CD4+ T cells (blue) and HIV-infected (HSA+) memory CD4+ T cells (red). Results are pre-gated on live, singlet CD3+CD8-CD45RO+CD45RA- cells. The proportion of uninfected and infected cells are indicated in blue and red, respectively. (TIF) [file ppat.1008450.s002.tif]

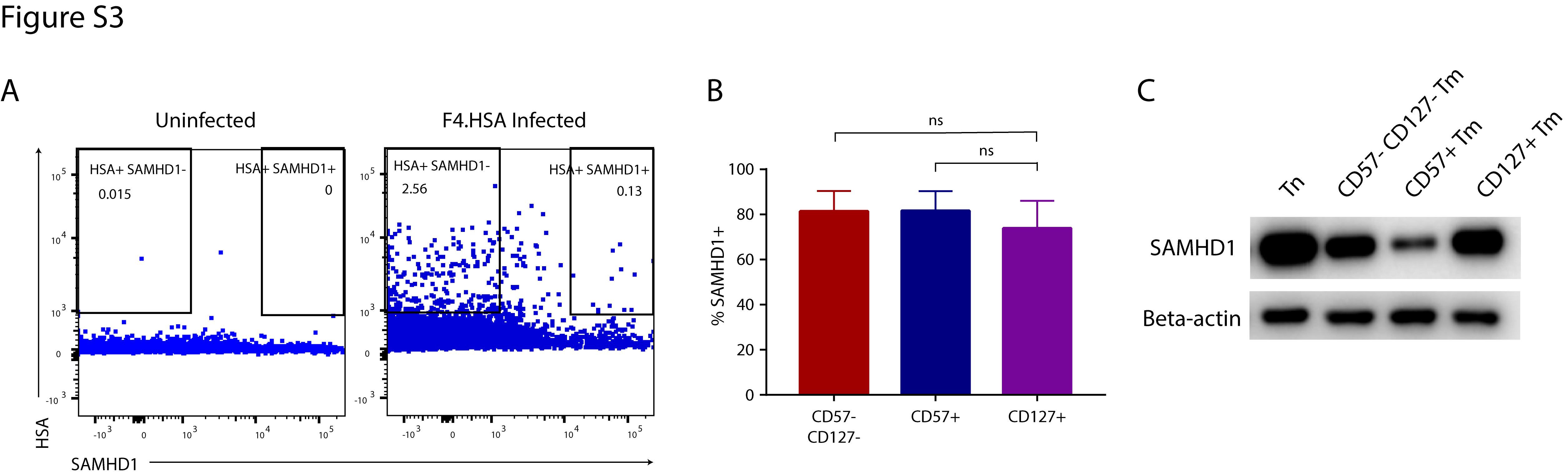

Supplement: S3 Fig — A) Validation of SAMHD1 antibody by flow cytometry. Unstimulated PBMCs were mock-treated or exposed for 3 days to F4.HSA. Depicted within the gates are the proportions of infected cells amongst the SAMHD1- and SAMHD1+ cells. The results demonstrate that SAMHD1- cells, which are permissive, harbor a higher proportion of infected cells than non-permissive, SAMHD1+ cells, as expected [3]. Results are pre-gated on live, singlet CD3+CD8-CD45RO+CD45RA- cells. B) Bar graph of the percentage of SAMHD1+ cells across the three indicated memory CD4+ T cell subsets from HLACs as determined by flow cytometry. Results are pre-gated on live, singlet CD3+CD8-CD45RO+CD45RA- cells. ns: non-significant as determined using a 2-tailed paired parametric t-test. C) SAMHD1 levels in the three Tm subsets from HLACs as determined by Western blot. Beta-actin served as a loading control. (TIF) [file ppat.1008450.s003.tif]

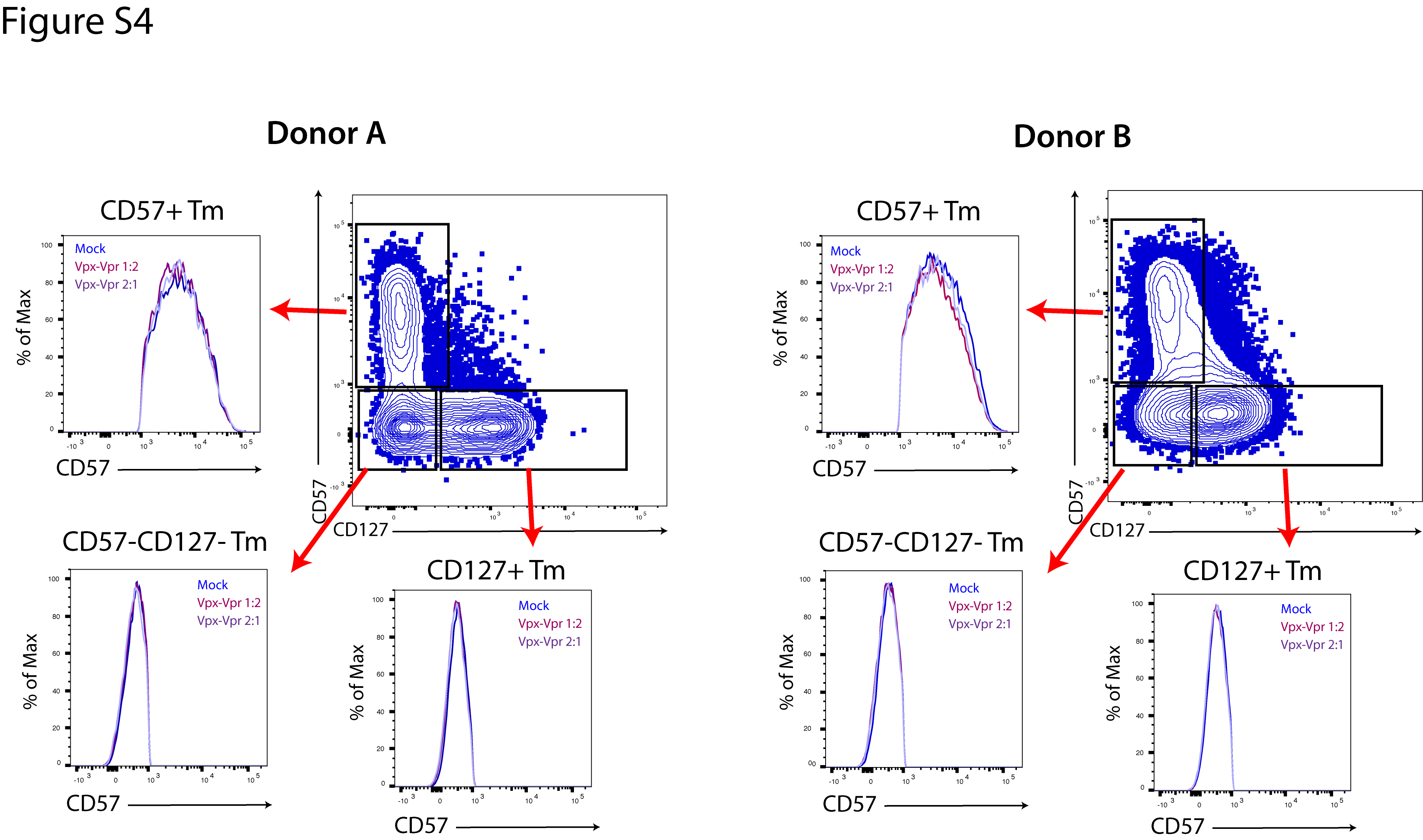

Supplement: S4 Fig — HLACs from two donors were sorted for CD127+, CD57+, and CD57-CD127-Tm cells as indicated in the contour dot plots. Cells were then mock-treated or exposed for 3 days to F4.HSA virions harboring Vpx at a ratio of 1:2 or 2:1 as indicated. Three days later, cells were monitored for cell-surface expression levels of CD57 by FACS. The data demonstrate that Vpx did not increase CD57 expression levels in any of the sorted Tm populations. (TIF) [file ppat.1008450.s004.tif]

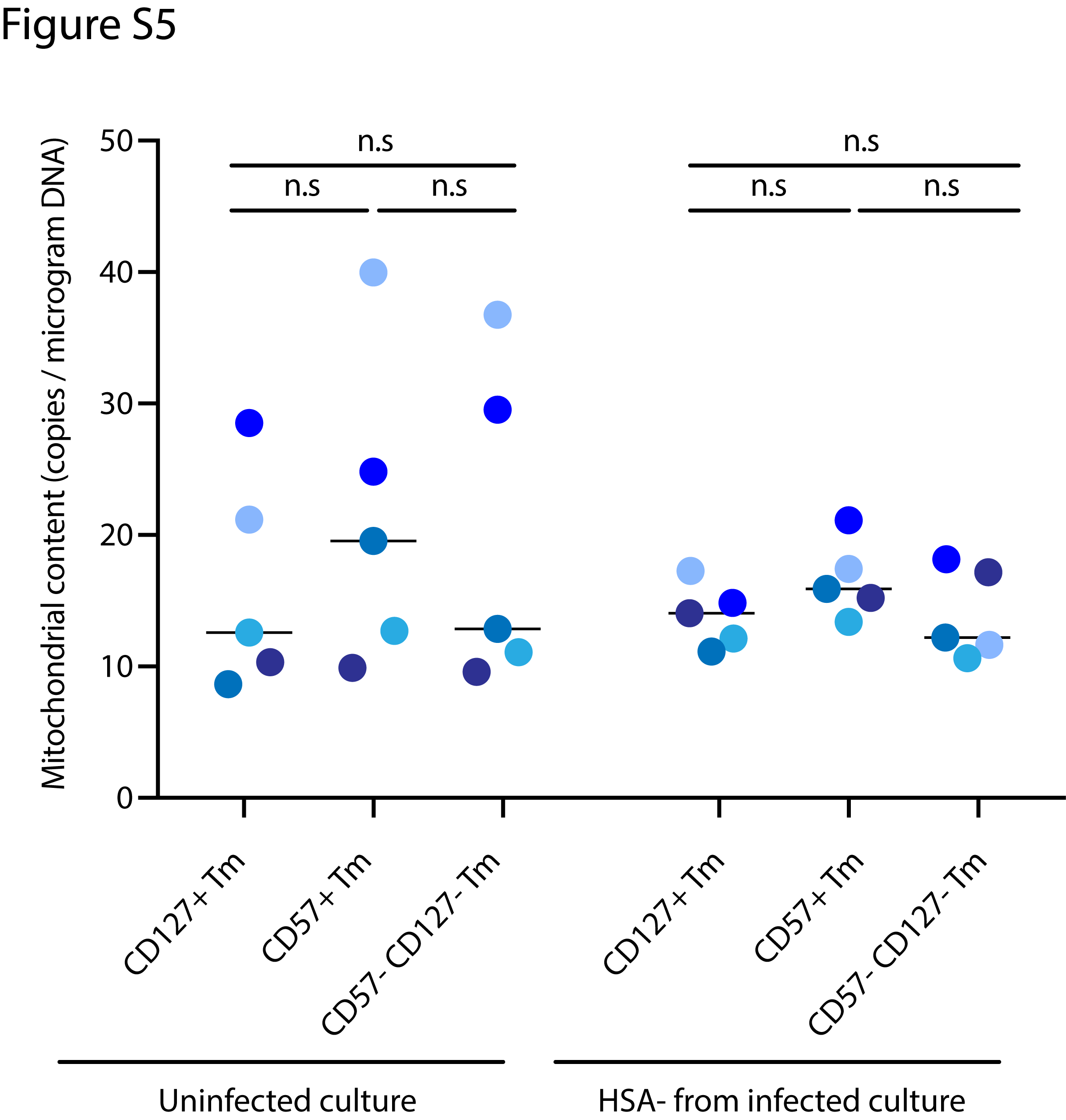

Supplement: S5 Fig — The indicated populations of cells were sorted from uninfected cultures (left) or HIV-infected cultures (right). Infected cultures were sorted on HSA- cells to eliminate productively-infected cells from the culture. Each donor specimen is indicated by a different color. n.s.: not significant, as determined by a one-way paired ANOVA using a Bonferroni posttest to account for multiple comparisons. (TIF) [file ppat.1008450.s005.tif]

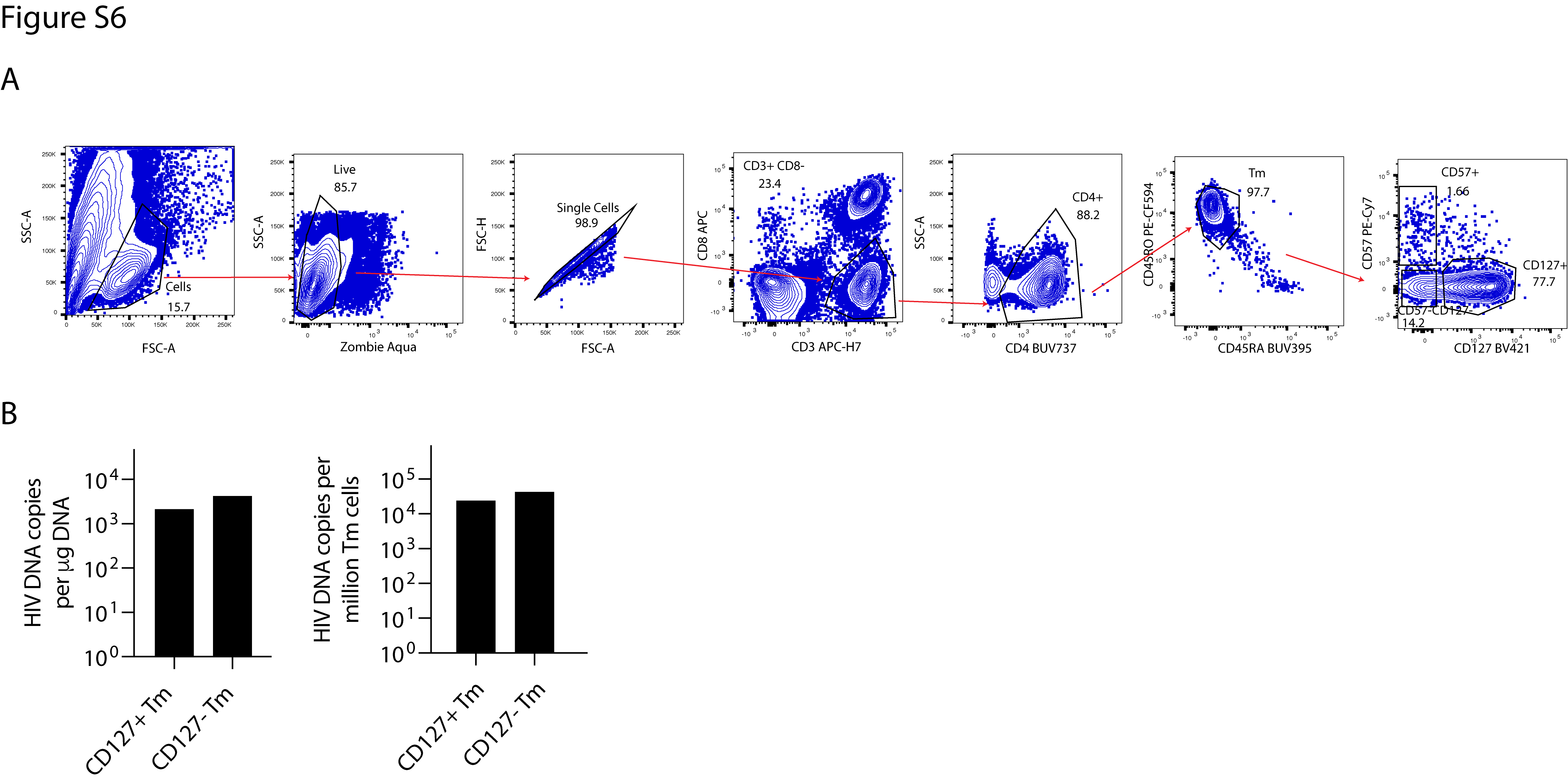

Supplement: S6 Fig — (A) Gut specimens were obtained via sigmoid biopsies from an ART-suppressed, HIV-infected individual treated during chronic infection. The specimens were processed into a single-cell suspension, and sorted for CD127+, CD57+, and CD57-CD127- Tm cells as shown. Because the recovery of CD57+ Tm cells was low, these cells were combined with the CD57-CD127- Tm cells to generate one single population of CD127- Tm cells. (B) The sorted CD127+ and CD127- Tm cells were assayed for the levels of HIV DNA by ddPCR. Data are reported as the HIV DNA copies per microgram of total DNA (left), or per million cells as determined by copies of the TERT housekeeping gene (right). (TIF) [file ppat.1008450.s006.tif]

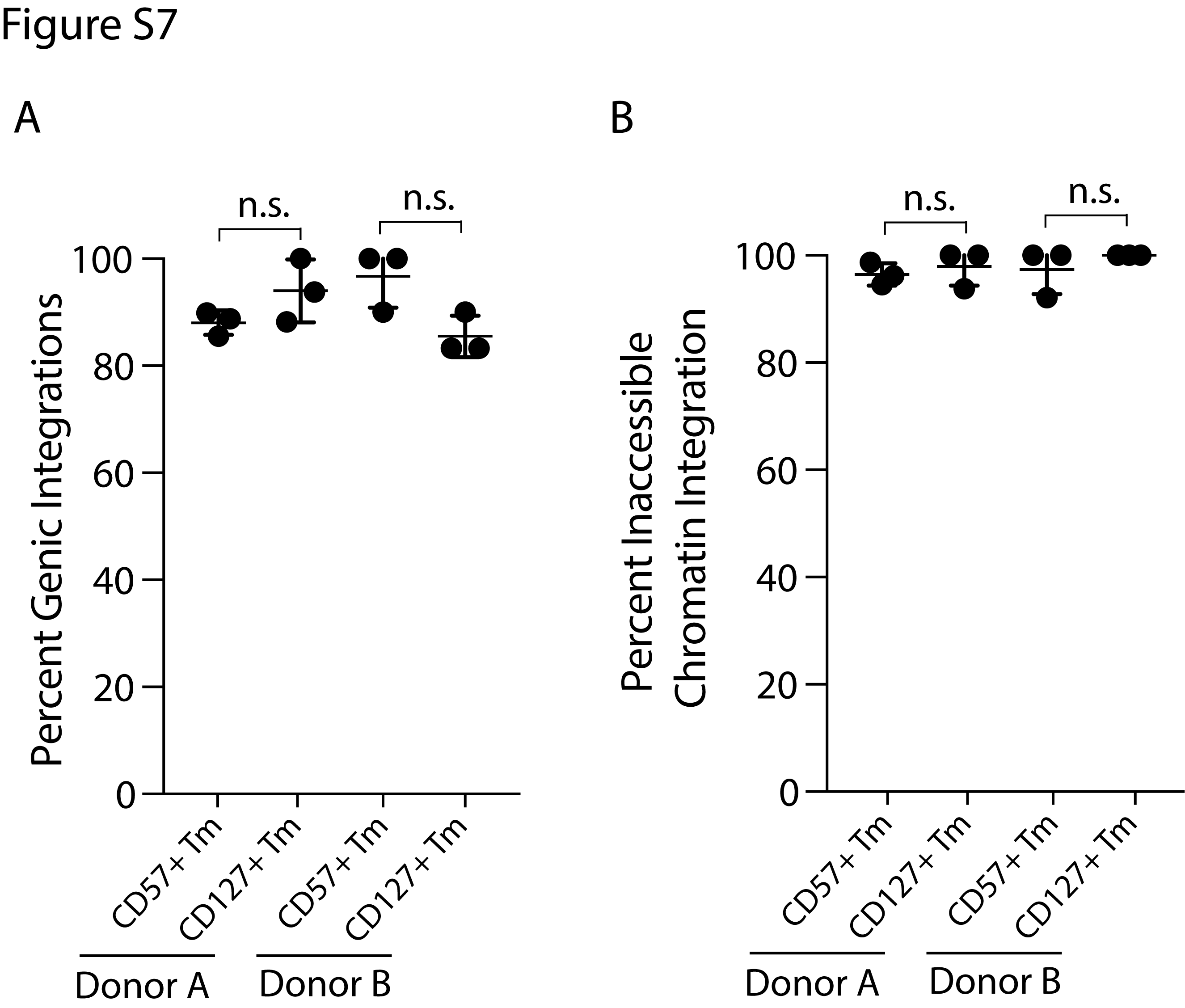

Supplement: S7 Fig — HSA- CD127+ Tm cells or HSA- CD57+ Tm cells were sorted from F4.HSA-infected cultures from two donors, and assessed for the genomic sites of HIV integration. The frequencies of viral integrations into genic regions of the human genome did not show a consistent trend of in-gene enrichment/depletion between the two subsets (A). Although there was a trend whereby the integration sites in CD127+ Tm cells were less frequently found in accessible regions of the chromosome as compared to in CD57+ Tm cells (as defined by publicly-available DNase-seq and ATAC-seq datasets from CD4+ T cells), this trend was not statistically significant (B). The three data points in each sample correspond to three sampling replicates from the same sorted specimen. Black horizontal lines between dots represent means, and standard deviations are shown as error bars. n.s.: non-significant, as assessed by 2-tailed unpaired student’s t-tests. (TIF) [file ppat.1008450.s007.tif]

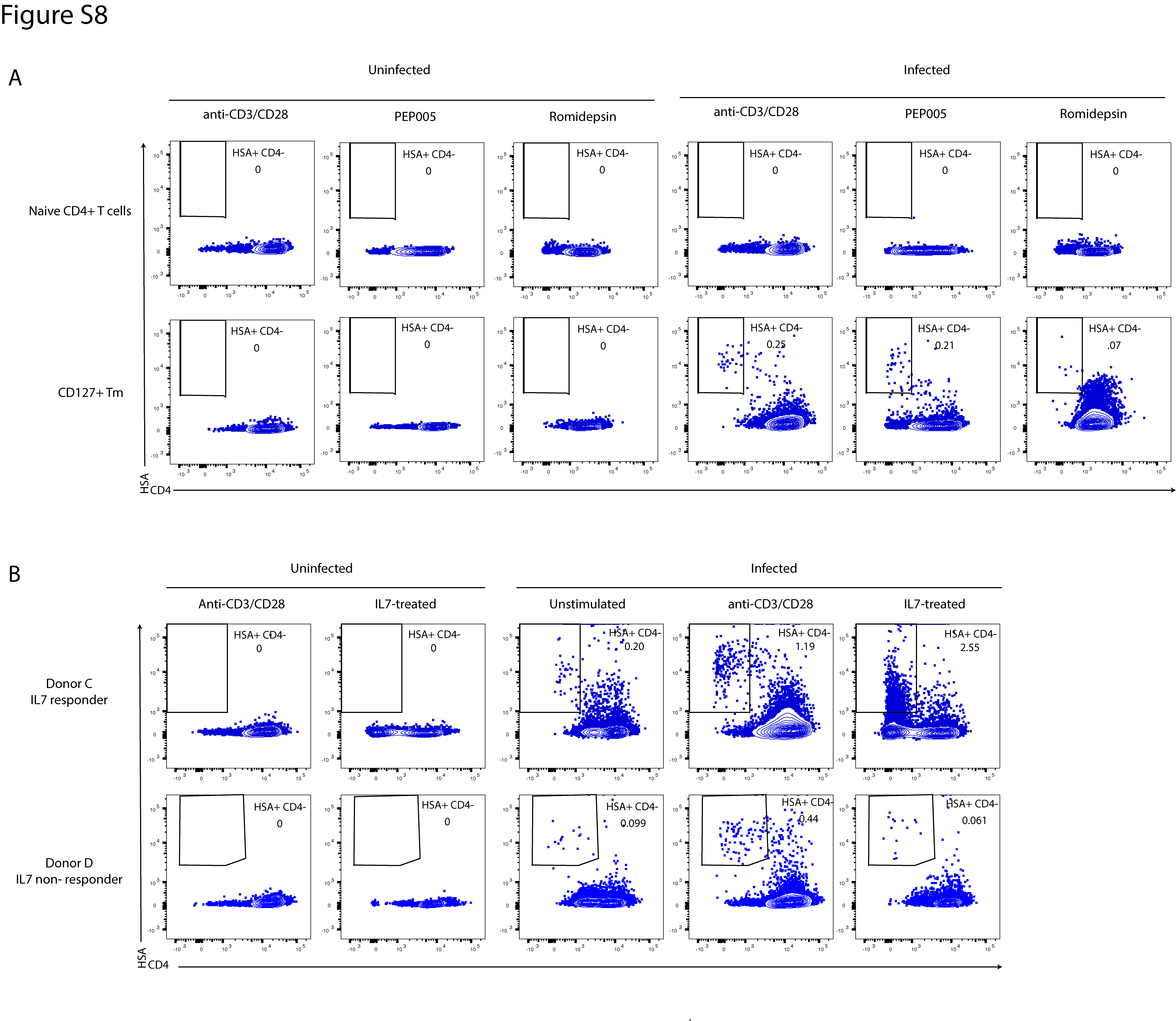

Supplement: S8 Fig — (A) HLAC cultures were mock-treated or exposed to F4.HSA and then sorted for HSA- CD127+ Tm or naïve CD4+ T cells, which were then cultured with media alone or in the presence of 12 nM of the ingenol PEP005 or 40 nM of the HDAC inhibitor romidepsin. Reactivation levels were compared to stimulation with anti-CD3/CD28. The proportion of infected (HSA+) cells that have downregulated cell-surface CD4 are indicated. Data are representative of one of two donors. (B) HLAC cultures from two donors were either mock-treated or exposed to F4.HSA, and then sorted for HSA- CD127+ Tm cells, which were then stimulated with anti-CD3/CD28 or 10 ng/ml IL7. The top row shows an example of a donor whose latently-infected CD127+ Tm cells reactivated in response to IL7 treatment, while the bottom row shows an example of a different donor whose CD127+ Tm cells did not respond in this manner. The proportions of infected (HSA+) cells that have downregulated cell-surface CD4 are indicated. (TIF) [file ppat.1008450.s008.tif]

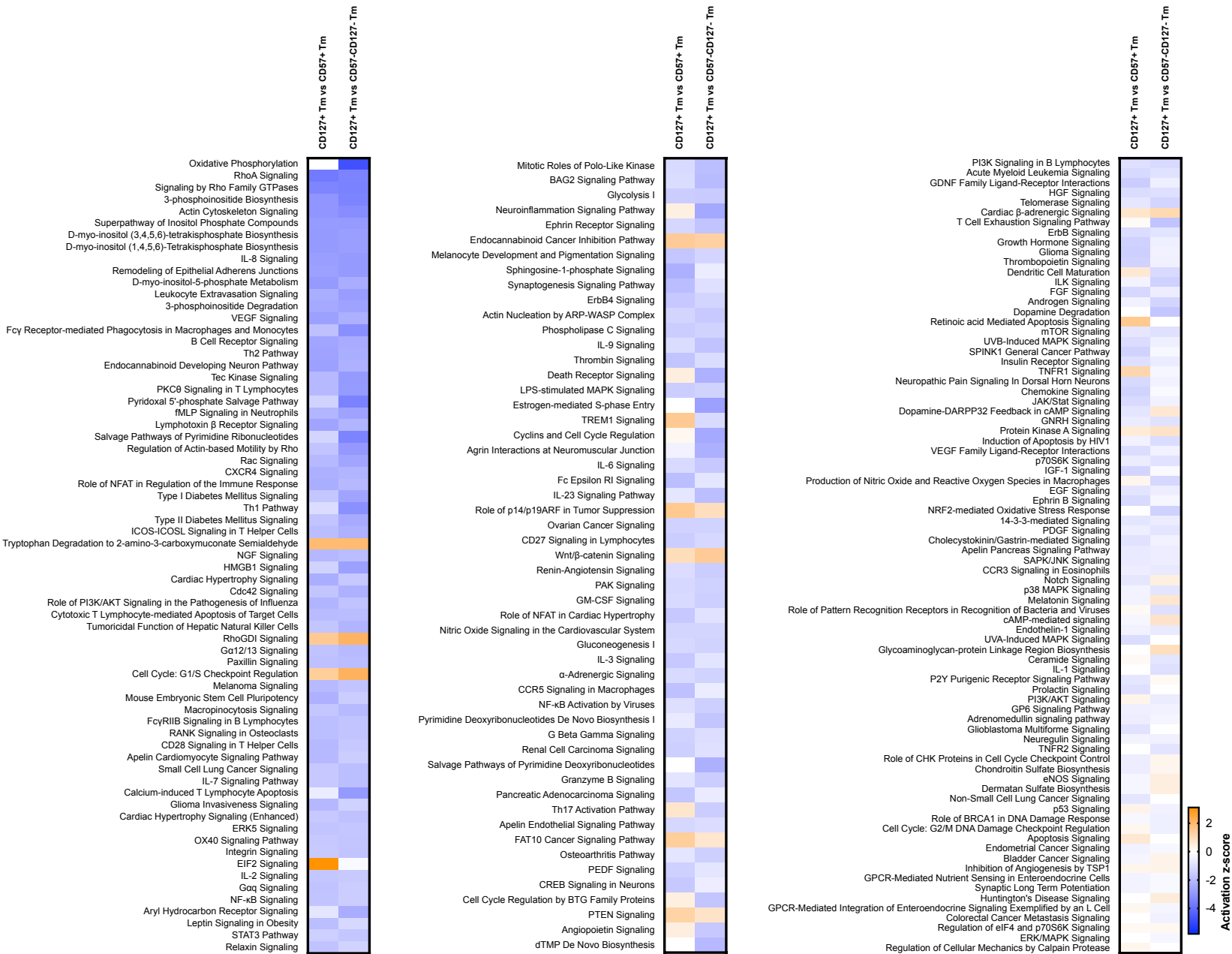

Supplement: S3 Table — Pathways are listed according to z-score, from top to bottom (highest to lowest absolute z-score) with a p-value cutoff filter of log101.3. Blue corresponds to pathways downregulated in CD127+ Tm cells, with the intensity of the blue corresponding to the extent of downregulation. Orange corresponds to pathways upregulated in CD127+ Tm cells. (PDF) [file ppat.1008450.s011.pdf]
