# Supplementary material for: Tissue memory CD4+ T cells expressing IL-7 receptor-alpha (CD127) preferentially support latent HIV-1 infection
Source: PLoS Pathog. 2020 Apr 30;16(4):e1008450. doi: 10.1371/journal.ppat.1008450 (PMC7192375; doi:10.1371/journal.ppat.1008450)
Supplement: Attachment — Submitted filename: Rebuttal.docx [file ppat.1008450.s012.docx]

**Point-by-point Rebuttal**

Dear PLOS Pathogens Editorial Team,

We thank you and the Reviewers for the thoughtful comments and for appreciating the significance of our study, and for encouraging a resubmission of the manuscript. Please find below our point-by-point response to the Reviewers’ comments *in italics*, followed by our responses.

**Reviewer #1**

Summary and Major Issues

*Since HIV-1 infects primarily residing in lymphoid tissues, the HLAC system is a significant advancement over models that utilize peripheral blood cells. As such, results emerging from this model could provide novel insights into the nature of cells harboring latent HIV, as well as the molecular mechanisms that underlie this phenomenon. While the studies described in the manuscript are intriguing, it still remains unclear whether CD127+ cells represent a significant reservoir of latent HIV infection in vivo.*

We thank the reviewer for recognizing that the HLAC system we described is “a significant advancement over models that utilize peripheral blood cells” and that “results emerging from this model could provide novel insights into the nature of cells harboring latent HIV, as well as the molecular mechanisms that underlie this phenomenon”. With regards to whether the CD127+ cells represent a significant reservoir of latent infection *in vivo*, this is of course extremely important to address, but tissue specimens from HIV-infected individuals are difficult to come by, and it is for this reason that almost all reservoir studies on people living with HIV have been conducted on blood specimens. However, in response to this comment, we have procured sigmoid biopsies from an ART-suppressed, HIV-infected individual. Memory CD4+ T cells expressing CD127 were sorted out and compared to their CD127- counterparts for levels of HIV DNA. We found that the two populations of tissue Tm cells both harbored high levels of HIV DNA. These new data are now presented as Fig. S6. Too few tissue cells after sorting were available to determine what proportion of these latent cells harbor intact or replication-competent viral genomes. These data nonetheless suggest that tissue-derived memory CD4+ T cells expressing CD127 are an HIV reservoir *in vivo*, and are consistent with the *in vitro* studies described herein.

*All in vitro models are biased one way or another, and the one utilized in this study is no exception. In this case, the bias is that resting (or minimally activated) cells are infected with HIV in the absence of exogenous activation. What is the evidence that in vivo HIV infects resting or minimally activated cells at frequencies comparable to infection of activated cells, thus justifying the extension of these conclusions to the in vivo setting? What is the evidence that this model reflects the in vivo situation more faithfully than model using exogenous stimulation?*

The value of the model we report here is the use of primary tissue cells, in contrast to blood cells used in other models. The fact that we do not need to stimulate these cells with exogenous factors is a benefit of this model, although we acknowledge that we do not have direct evidence that exogenous stimulation of primary cells prior to establishing latency would be less reminiscent of the *in vivo* situation than a system where no exogenous stimulation is required. That being said, we do know that stimulation of the tissue cells in our model changes their phenotype (the original Fig. S1, now Fig. S2), suggesting that exogenous stimulation of these cells is likely to change them in ways that make them less reminiscent of their original *in vivo* state. Furthermore, our new patient data (Fig. S6) suggest that our *in vitro* system characterizes a population of tissue cells that serve as an *in vivo* HIV reservoir. In order to not imply that protocols that do not use exogenous stimulation reflect the *in vivo* situation more faithfully than protocols that do, we have modified our statement on this topic to state that the lack of exogenous stimulation, and lack of requirement for a prolonged period of culture, makes the system easy to use and is a benefit of the model.

*Since CD57+, CD127+ and double negative cells present different baseline levels of transcriptional activity (even in the absence of HIV infection) and present different degrees of cell quiescence/activation, it is expected that HIV will integrate with different patterns of genomic distribution in the three subsets. In particular, since CD57+ and double negative cells are more transcriptionally active (i.e. have a much more open chromatin structure) than CD127+ cells, HIV is likely to integrate in euchromatic regions of these subsets more frequently than in CD127+ cells. Have the authors considered whether—and, if so, how—this will influence baseline and re-stimulated HIV expression in the three subsets? Is it possible that in this specific in vitro model, increased frequency of HIV integration in heterochromatic regions also plays a role in the deeper state of latency in CD127+ cells? If so, how does the question above about the physiological relevance of HIV infection of resting (or minimally activated) cells impacts the results?*

The Reviewer brings up an interesting idea, that HIV may integrate into different genomic locations within the CD127+ Tm cells relative to other Tm subsets that have a higher propensity to become productively-infected. In response to this comment, we conducted experiments to whereby latently-infected CD127+ Tm cells and their CD57+ Tm counterparts were sorted and integration site analysis was performed. We identified a total of 1,207 unique integration sites from our analyses. Because euchromatin tends to be gene-rich while heterochromatin tends to be gene-poor (Tamaru et al 2010, Genes Dev 24(14):1465; Solovei et al 2016, Curr Opin Cell Biol 40:47), we first mapped our integration sites to genic versus non-genic sites. Our results revealed that the virus integrated predominantly into genic regions, and not non-genic regions. Moreover, there was no consistent trend as to whether the sites of integration within the CD127+ Tm cells were more in genic regions as compared to in the CD57+ Tm cells. To more directly assess integration into euchromatin vs. heterochromatin, we mapped our integration sites against publicly-available datasets of chromatin accessibility. We combined DNase-seq data from 13 samples of CD4+ T cells from the publicly available ENCODE project and ATAC-seq data from 114 samples of CD4+ T cells from a published study (Gate et al., Nat Genet 2018, 50(8):1140) and designed a custom script to determine whether our integration sites overlapped with genomic intervals classified as peaks (see revised Methods). These results demonstrated a suggestive trend whereby integration in the CD127+ Tm subset tended to preferentially occur in more inaccessible regions of the genome when compared to the CD57+ Tm subset, but the data were not statistically significant. These data suggest that the propensity for CD127+ Tm cells to become latently-infected is unlikely to be driven primarily by HIV preferentially integrating into heterochromatic regions in these cells. Rather, other aspects of the intracellular environment of these cells, such as the availability of transcription factors to drive LTR-driven gene expression, may be responsible. These new data are now presented as Figure S7.

*The role of “chromatin openness” at the time of virus infection and integration is reflected in the evidence that even after re-stimulation with anti-CD3/CD28, the CD127+ subset expresses HIV at significantly lower levels than the other two (see below). While, the studies reported here show that CD127+ cells present much lower levels of basal transcriptional activity (i.e. at the resting state), the studies do not show—nor is it known—whether this is also the case following cell stimulation (i.e. at the activated state). Therefore, the lower levels of HIV expression following re-stimulation (and perhaps to some extent even at baseline in unstimulated cells) may be the consequence of preferential integration in heterochromatic regions of the genome. This ought to be addressed.*

We would like to point out that latently-infected CD127+ Tm in fact reactivated more efficiently than latently-infected CD57+ Tm and CD57-CD127- Tm. This was not shown in the original submission but is in the revised manuscript (please see the revised Figure 5 and further explanation below). As discussed above, we have also looked at the genomic locations of HIV integration in the CD127+ Tm cells, and did not find that HIV has a strong preference for integration into heterochromatic regions of CD4+ T cells in the CD127+ Tm cells relative to other Tm cells.

*Based on the legend to Fig. 5, panel B of that figure shows the copy number of HIV transcripts normalized to total number of CD127+ or total memory cells rather than to the number of infected cells in the total memory and CD127+ memory subsets. Since CD127+ cells contain integrated HIV at frequencies ~2 logs higher than CD57+ and double negative cells (see Fig. 3E), HIV transcription in CD127+ is consequently up to 100-fold (2 logs) lower on a “per infected cell” basis compared to the other two populations.*

The reviewer brings up a good point that if CD127+ Tm harbor markedly higher frequencies of integrated HIV relative to the other populations, then the amount of HIV transcription would be lower in these cells on a “per infected cell” basis. It turns out that the CD127+ Tm do not always have higher levels of integrated HIV DNA relative to the other populations. Although that is sometimes observed (as exemplified in Figure 3D), other times the levels of HIV DNA were higher in the other populations. Importantly, however, what is always consistent is that the amount of HIV DNA relative to the number of productively-infected cells is always disproportionately high in the CD127+ Tm, as exemplified in Figure 3E. To clarify this point, we have modified both the manuscript (Lines 211-216 in clean version) and the legend for Figure 3E to reflect this. That being said, we agree with the reviewer that it important to normalize the transcription data to the number of infected (HIV DNA+) cells. Therefore, in the revised Figure 5B, we now show the data both normalized by total number of cells in the subset, as well as by amount of HIV DNA in that subset. This way of normalizing the data does not change the original conclusion, that the CD127+ Tm cells harbor some HIV transcription.

*At the same time, following re-stimulation the frequency of HSA+ cells in the CD127+ population is only 6-fold greater than in the total memory population. Again, the CD127+ population contains integrated HIV at frequency 100-fold higher than the other two memory populations, therefore a much smaller fraction of latently infected CD127+ cells can be induced to reactivate HIV compared to CD57+ and double negative cells. In light of these conclusions, and in consideration of the fact that these studies utilized an in vitro model whereby cells are re-stimulated merely 3 days post-infection, it could be argued that CD127+ memory cells are indeed in a state of deep quiescence and the virus they harbor is in a state of deep latency. How do the authors define the concept of “deep latency”?* *Since this concept is not quantifiable and is not univocally defined in the literature or in the present manuscript, it is recommended that the authors revise their conclusions in this regard accordingly.*

We agree that we did not clearly define what was meant by “deep latency,” and that since this concept is not quantifiable, we should revise our conclusions in this regard. We have there removed all mention of “deep latency” and instead focused on the discussion on whether latently-infected CD127+ Tm can be reversed from latency.

Minor Issues

*Reviewer #1: The quality of the figures is very poor. Although the final figures will have a much greater resolution, these images should have sufficient resolution to be readable by the Reviewers. Some panels cannot be read at all (Fig. 3B-D; Fig. 4B-F; Fig. 5B and 5C). This is not acceptable.*

The resolution of the figures in the PDF is poor because the Plos Pathogens submission process reduces the resolution of these images when it compiles the final PDF. The original (high-resolution) versions of all the figures are available by clicking at the link in the top right of each figure within the compiled PDF, which will download a high-resolution tiff file of the corresponding figure. We have confirmed that the links work in the original submitted PDF, and in the newly generated one for the resubmission.

**Reviewer #2**

Summary and Major Issues

*The entire paper's data is from in vitro HIV infections of human lymphocyte aggregate cultures (HLACs) with no validations from HIV+ people samples, which is a major drawback. Especially, since they are eluding to the idea that in vivo an HIV reservoir could be present in CD127+ memory CD4 T cells.*

We agree that it is important to assess to what extent CD127+ Tm represent a reservoir *in vivo*, and had not done so in the original manuscript as tissue specimens from HIV-infected individuals are difficult to come by, which is also the reason why most HIV reservoir studies using *in vivo* specimens have analyzed only blood specimens. We feel that is why *in vitro* tissue models of HIV latency are important for the field. However, in response to this comment, we have procured sigmoid biopsies from an ART-suppressed, HIV-infected individual. Memory CD4+ T cells expressing CD127+ were sorted out and compared to their CD127- counterparts for levels of total HIV DNA. We found that the two populations of cells from gut harbored similar levels of DNA, data that are now presented as the new Fig. S6. Too few tissue cells after sorting were available to determine what proportion of these latent cells harbor intact or replication-competent reservoir cells. Nonetheless, these data demonstrate that tissue-derived memory CD4+ T cells expressing CD127 are an HIV reservoir *in vivo*, and are consistent with the *in vitro* studies described herein.

*1. In Figure 1 the CD127+ Tmem population is very small in comparison to CD57+ Tmem cells. Since there are not that many CD127+ CD4+ T cells to begin, why isn’t the poor infection due to having fewer of these target cells compared to the CD57+ Tmem cells that are much more abundant? The authors should sort and infect equal number of CD127+ Tmem and CD57+ Tmem cells to address availability of these cells to be infected with HIV.*

The donor presented in Figure 1A was one that had a smaller population of CD127+ Tm than CD57+ Tm, but this was not always the case and the relative frequencies of these two memory populations was donor-dependent. Importantly, CD127+ Tm infection rates were extremely low relative to those in CD57+ Tm, even when the abundance of CD127+ Tm cells was higher than the abundance of CD57+ Tm. Two examples of this are now shown in the newly added Figure S1. These data demonstrate that the poor infection rate in CD127+ Tm is not due to their being less abundant, an aspect that is now also discussed in the manuscript (Lines 127-130 in clean version).

*2. The experiments in Figure 2 and supplemental figure S2 looking at SAMHD1 restriction as a mechanism for blocking mHSA expression does not fully address the authors hypothesis and conclusion. The authors should sort CD127+ Tmem cells, CD57+ Tmem cells, and double negative cells from the HLACs and measure SAMHD1 phosphorylation of threonine 592. Especially since signaling through IL7R induces SAMHD1 phosphorylation of T592.*

We had shown in the original Figure S2C that SAMHD1 phosphorylated at threonine 592 was present in HLACs at levels too low for detection by Western blot (whereas our positive control of THP1 cells expressed phosphorylated SAMHD1 readily detectable by Western blot). These results had suggested that all 3 of the Tm populations present in HLACs should express very low levels of phosphorylated SAMHD1. To confirm these findings, we went ahead and sorted these populations as the reviewer requested, and confirmed that phosphorylated SAMHD1 was not detectable in CD127+ Tm cells, CD57+ Tm cells, or CD57- CD127- Tm cells even when the blot was overexposed. These data are now presented as the new Fig. 2A. Because these new data are redundant with our previous Fig. S2C data that phosphorylated SAMHD1 is not readily detectable in bulk HLACs, we have removed those old data and replaced with the new ones described here.

*In panel B of figure 2 the data can be interpreted as Vpx-Vpr in HIV-infected CD57 negative cells causes these cells to express CD57, therefore there is an increase in the frequency of CD57+ HIV-infected cells. The authors should address if Vpx-Vpr is activating CD57 expression in contrast to Vpx-Vpr allowing CD57+ Tmem cells to be more permissive to HIV.*

To demonstrate that Vpx-Vpr wasn’t increasing CD57 expression in CD57- cells, we sorted CD127+ Tm, CD57+ Tm, and CD57-CD127- Tm and treated these cells with virions harboring Vpx-Vpr. As demonstrated in the new Figure S4, Vpx did not increase expression in any of the sorted populations, demonstrating that Vpx-Vpr does not activate CD57 expression.

*3. ddPCR experiments in Figure 3 should normalize the integrated HIV DNA data with a region in the human genome. Normalizing to mitochondria DNA can introduce a source of bias. The authors did not demonstrate CD57+ Tmem. CD127+ Tmem, and double negatives have equal levels of mitochondria DNA, which explains why they chose this DNA for normalization. They should report how many cells were sorted for each population and plot the number of integrated HIV to number of input cells determined by the amplified region of the human genome.*

We had used mitochondrial DNA for normalization because it is one of the methods that has been used to normalize for levels of HIV DNA (e.g., Reinhard et al 2014, Retrovirology 11:12, and Butler et al 2001, Nat Med 7:5:631). Both of these references are now cited in the Methods description. Furthermore, we prepared a new figure demonstrating that the sorted Tm specimens used in the original analyses had equivalent levels of mitochondrial DNA (the new Fig. S5), validating this method of normalization for our datasets.

*A second source of bias is the calculation of integrated HIV DNA to infection rate. The CD127+ Tmem population have a very low frequency of infection therefore a signal of integrated HIV DNA gets amplified when divided to infection frequency.*

We had reported the amount of integrated HIV DNA relative to infection rate to make the point that CD127+ Tm always exhibited a disproportionately high amount of HIV DNA relative to how much productive infection was observed in these cells. Of note, the CD127+ Tm did not always have the highest amount of integrated DNA. To not be misleading in this aspect, we have now added a statement that although the levels of integrated HIV DNA in the CD127+ Tm varied from donor to donor, the levels of integrated HIV DNA relative to infection rates were always disproportionately high in the CD127+ Tm cells compared to the other two subsets of memory CD4+ T cells (Lines 211-216 in clean version).

*4. Figure 5 should have experiment that shows the frequency of infection without activation with anti-CD3/CD28 beads. How many cells that are CD127+CD57-CD4+mHSA- spontaneously express mHSA after three days without stimulation? The authors should also perform the same experiment on CD57+ Tmem cells. Will you also get latently HIV-infected cells from CD57+CD127-CD4+mHSA- cells?*

We thank the reviewer for making this suggestion and agree that these additional data are informative and important. We had included these other conditions in our original experiments but did not include the data in the originally submitted manuscript. We in fact had tested all three populations of Tm cells (CD127+ Tm, CD57+ Tm, and CD57-CD127- Tm) under both stimulated and non-stimulated conditions. The full set of data are now shown in the new Figure 5C. As can be seen in the figure, although in the HSA- CD127+ Tm cells there is some spontaneous HSA expression after 3 days without stimulation (0.095%), this rate is more than ten-fold lower than the amount of HSA expression following stimulation (1.06%). The latently-infected CD57+ Tm cells were also reactivatable. The sorted HSA- CD57-CD127- Tm exhibited similar levels of reactivated cells as the stimulated HSA- CD57-CD127- Tm, suggesting high levels of spontaneous reactivation in this population. These data are now discussed in Lines 321-324 in the clean version of the revised manuscript. Importantly, these data are all consistent with the original conclusion that latently-infected CD127+ Tm are reactivatable upon anti-CD3/CD28 stimulation.

Minor Issues

*There also appears to be a technical flow cytometry staining issue in supplemental figure S2 panel A. The staining does not look to be specific. It is known that SAMHD1 is expressed in every cell, so this data does not add any value to the overall hypothesis of SAMHD1's role in CD127+ Tmem cells.*

There was indeed some non-specific staining of cells with the SAMHD1 antibody as assayed by flow cytometry. We therefore additionally probed SAMHD1 levels in sorted populations of Tm cells by Western blot. These new data, now presented as Fig. S3C, revealed relatively low levels of SAMHD1 expression in the three Tm populations as compared to the levels in naïve CD4+ T cells, as expected due to the ability of naïve CD4+ T cells to restrict HIV infection. The levels of SAMHD1 expression in the non-permissive CD127+ Tm cells were similar to those in the permissive CD57-CD127- Tm cells. Together, the flow cytometry and Western data suggest that the CD127+ Tm cells do not express significantly higher levels of SAMHD1 than their permissive Tm counterparts.

**Reviewer #3**

Summary and Major Issues

*It is interesting that these cells are maintained in an aggregate culture system. There are opportunities to examine how this microenvironment might influence HIV-1 infection and expression. Experiments separating out cells, determining if there is a role for the IL-7R, adding in blood derived CD127+ cells are some experiments that could maybe address the role of the supporting cells in the establishment of latency.*

We thank the reviewer for suggesting to investigate a direct role for IL7R in maintaining latency in the tissue CD127+ Tm cells. To address this, we have conducted new experiments where we have assessed the ability of IL7 to reactivate latently-infected CD127+ Tm cells. These experiments revealed that IL7 could reactivate latently-infected CD127+ Tm cells, but the effect was donor-dependent. Interestingly, when IL7 treatment reactivated latent HIV, the treatment also downregulated expression of cell-surface CD4 independent of infection. These new results are now presented as Fig. S8, where we show an example of an IL7 responder (where IL7 reactivated latently-infected CD127+ Tm cells) and a non-responder (where IL7 did not reactivate latently-infected CD127+ Tm cells). With regards to the suggestion to add in blood-derived CD127+ Tm cells, these experiments are complicated by the fact blood CD127+ Tm are extremely efficiently productively-infected unlike their tissue counterparts (the original Fig. S1, now Fig. S2 in the revision). Furthermore, they are phenotypically distinct from their tissue counterparts in that they express high levels of CD57, and therefore it’s unclear whether we would classify them as being more closely related to the CD127+ Tm or the CD57+ Tm in tonsils. We have therefore focused our investigation of microenvironment on the role of IL7 and IL7R, and the effects of other potential LRAs (see below).

*2. Testing to see if latent infection in CD127+ cells could be induced by different reversing agents would provide insights into if these cells are in general easily reversed or if there maybe blocks to certain pathways. For example, it might be predict that HDACi would have modest effects on inducing HIV in these cells since they are expressing HIV mRNA whereas activating signals cascades upstream of NF-Kb or NFAT, might facilitate transcription and reverse the splicing defect.*

In response to this excellent suggestion, we have now tested the ability of the ingenol (PKC agonist) PEP005 and the HDAC inhibitior romidepsin to reactivate latently-infected CD127+ Tm cells. These results were compared to reactivation by anti-CD3/CD28. In line with what the reviewer postulated, the PKC agonist, which acts upstream of NFkB/NFAT, did indeed promote reactivation by HIV, in contrast to the HDAC inhibitor which did not reactivate the latently-infected CD127+ Tm cells. These new data have been added to the manuscript as Fig. S8.

*3. Was HIV-1 RNA observed in the RNA-seq experiments?*

The RNAseq experiment analyzed uninfected CD127+ Tm, CD57+ Tm, and CD57-CD127- Tm, as analyzing infected populations of these cells would be complicated by the ability of HIV to remodel or elicit innate immune sensing pathways in these cells. Therefore, no HIV RNA was observed in these cells.

Minor Issues

*Most of the SAMHD1 related data are presented as supplemental. This finding was presented as an important observation and should be presented in the main text.*

In our original submission, our SAMHD1 data were presented in the form of one main figure (the original Fig. 2) and one supplemental figure (the original Fig. S2). We have now moved most of the original Fig. S2 data as well as new Western blot data demonstrating no difference in phospho-SAMHD1 expression in the Tm subsets to the main figure (the new Fig. 2), We have kept as supplementary the data demonstrating no difference in SAMHD1 expression between the Tm subsets. This rearrangement presents important parts of the SAMHD1 data within the main figure.
