# Supplementary material for: Tissue memory CD4+ T cells expressing IL-7 receptor-alpha (CD127) preferentially support latent HIV-1 infection
Source: PLoS Pathog. 2020 Apr 30;16(4):e1008450. doi: 10.1371/journal.ppat.1008450 (PMC7192375; doi:10.1371/journal.ppat.1008450)
Supplement: Attachment — Submitted filename: Rebuttal.docx [file ppat.1008450.s013.docx]

**Point-by-point Rebuttal**

Dear PLOS Pathogens Editorial Team,

We thank the Reviewers for the thoughtful comments and are delighted to hear that the manuscript will likely be accepted for publication. We have modified the manuscript in response to the additional comments from this second round of review. Please find below our point-by-point response to the comments from Reviewers 2 and 3 *in italics*, followed by our responses.

**Reviewer #2**

Major Issues

*1. The experiments of supplemental figure 7 can be misleading. DNAseq and ATACseq experiments are biased towards detecting open DNA in promoters and enhancers. There are no reports that HIV integrates into these genomic elements. The data presented cannot be interpreted as “HIV more frequently integrated into closed, inaccessible regions…” and then end with “they [CD127+ cells] do not markedly favor integration of HIV into closed regions of the genome.” This result section has to be re-interpreted and re-written. The discussion portion of this conclusion also has to be re-written to address what is known about the epigenetic makeup of open/closed promoters and the corresponding adjacent gene bodies.*

We apologize for the confusing way this results section was written. The latter comment that the CD127+ cells do not markedly favor integration of HIV into closed regions of the genome was meant to emphasize the term “markedly,” and was referring to the fact that we only observed a trend (and not a statistically significant result) that HIV more frequently integrated into inaccessible regions in the CD127+ Tm cells as compared to the CD57+ Tm cells. We agree that the two phrases seem contradictory, and therefore deleted the latter sentence, and better emphasized the fact that we saw a non-significant trend in our results (Lines 298-301 in clean version).

In the Discussion section of the previous draft, we had simply commented that viral integrations in the CD127+ Tm cells mapped to both accessible and inaccessible regions of the human genome. In response to the Reviewer’s request to expand on this discussion, we have now added a new section (Lines 396 – 408 in clean version) that discusses the accessible regions (as defined by DNAse-seq and ATAC-seq data) being biased towards promoter regions, and our finding that latently-infected cells harbored provirus primarily integrated into inaccessible regions and gene bodies.

*2. Figure 5 panel C shows higher or equal level of productive infection without stimulation in the CD127+ cells in comparison to CD57+ cells. I thought the CD127+ cells were not becoming productively infected but instead undergoing latency. In addition, the stimulation of CD127+ and CD57+ cells are resulting the same level of productive infection, there is an 11-fold increase HIV infection from unstimulated to stimulated. Is this data representative?*

As highlighted in panel A of Figure 5, these experiments entailed sorting various populations of HSA- cells from the infected cultures. Because cells expressing the LTR-driven reporter HSA were removed during this sorting, most of the productively-infected cells should have been removed. Therefore, in Fig. 5C the slightly higher infection one sees in the unstimulated CD127+ Tm cells relative to CD57+ Tm cells corresponds to a slightly higher rate of spontaneous reactivation in this donor, and not to direct productive infection. Of note, in some donors the spontaneous reactivation rate was higher in the CD127+ Tm cells, while in others it was higher in the CD57+ Tm cells. To clarify that the infected cells we are showing here correspond to reactivated cells, we have added into the figure legend the sentence “The infected cells in the non-activated samples are expressing HSA due to spontaneous reactivation of the sorted HSA- cells, while those in the activated samples correspond to stimulation-induced reactivation.”

With regards to how representative the 11-fold increase in infection rates from unstimulated to stimulated CD127+ Tm cells is, in the original submission we had conducted the experiment in two donors, with the induction being 11.2-fold in the first donor and 5.5-fold in the second. We have since conducted the experiment on two additional donors, one exhibiting a 4.4-fold induction and the other 6.0-fold induction. To reflect these findings, we have added into the figure legend the following sentence: “Shown are results of one of four representative donors, with the average induction in infection rates, in CD127+ Tm cells, between the non-activated and activated cultures being 6.8-fold (range 4.4 – 11.2-fold).”

*3. The authors never address the intactness or replication-competency of the HIV provirus in either CD127+ or CD127- memory CD4 T cells in their in vitro work. They should mention that their findings have a limitation in terms of understanding if there are plenty of intact replication-competent proviruses in CD127+ or CD127- cells to merit these populations harboring the latent replication-competent HIV reservoir in cART.*

Our finding that latently-infected CD127+ Tm cells can be reactivated demonstrates that the provirus in these cells is likely intact and replication-competent, since they can be induced to express the LTR-driven reporter HSA. Consistent with this, our integration site analysis, which used near-full-genome primers, also found that the vast majority of our integrants were intact. To make the point that our *in vitro* system produces mostly intact integrants while acknowledging that our Alu-Gag ddPCR assay to quantitate integrated provirus did not distinguish between intact and defective genomes, we have added the sentence “Although the Alu-Gag ddPCR assay cannot distinguish intact from defective provirus, given that our *in vitro* culture system is relatively short-term and not subject to immune-mediated pressures, it is likely that most of the sequences we are detecting are intact” to Lines 218-221 in the clean version.

Minor Issues

*1. Figure 3, panel C the top flow plot shows the three populations that are being sorted, however the x- and y-labels are incorrect.*

We apologize for this labeling mistake, and thank the reviewer for catching this. The figure has now been corrected.

*2. The primary conclusion of Figure 3 is that CD127+ memory CD4 T cells support latent HIV infection, because there are higher levels of integrated HIV copies in the CD127+ cells when compared to CD57+ or CD57-CD127- cell. However, Figure 2, the CD57+ and CD57-CD127- cells have a higher level of productive infection that suggests the productive infection is from non-integrated HIV proviruses since you are showing very little integrated HIV DNA in these cells. These results have to be better explained.*

The CD57+ and CD57-CD127- Tm cells do not always harbor less integrated DNA than the CD127+ Tm cells. Figure 3E demonstrated that the levels of integrated HIV DNA relative to productive infection rates was disproportionately high in the CD127+ Tm cells relative to these other two subsets; this occurred sometimes when the CD57+ and CD57-CD127- Tm cells had higher levels of integrated HIV DNA than the CD127+ Tm cells. To not give the inaccurate impression that there is in general little integrated HIV DNA in the CD57+ and CD57-CD127- Tm cells (and that productive infection would be coming from non-integrated HIV proviruses), we have modified the results section to state the following “Repeating this experiment with cells from a total of 5 independent donors revealed that the levels of integrated HIV DNA in the CD127+ Tm cells varied from donor to donor, and were sometimes higher than that in CD57+ and CD57-CD127- Tm cells and sometimes lower. Importantly, however, the ratios of integrated HIV DNA to productive infection rates as defined by HSA expression were always disproportionately higher in the CD127+ Tm cells than in the other two subsets of memory CD4+ T cells (Fig. 3E).” (Lines 213-218 in clean version).

*3. The conclusion from the data showing SAMHD1 does not impact productive infection of CD127+ cells doesn’t fully address the experiments addressing the role of SAMHD1 restricting HIV infection. Did the Vpx-Vpr infections of CD127+ cells lead to an increase of integrated HIV DNA in these cells?*

The point of the Vpx-Vpr experiments was to demonstrate that diminishing the activity of SAMHD1 does not rescue productive infection of CD127+ Tm cells, which suggested that SAMHD1 was not acting to restrict HIV infection at the pre-integration stage as might have been expected from its ability to deplete the intracellular pool of dNTPs needed for reverse transcription. This was what prompted us to then examine whether these cells can become latently infected. Whether abrogating SAMHD1 activity might increase integration of the HIV provirus without increasing productive infection in the CD127+ Tm cells was not examined and we feel is beyond the scope of this manuscript.

*4. Their gut biopsy of ART-suppressed HIV-infected individual CD127+ and CD127- memory CD4 T cells had detectable HIV DNA. That mean that both populations can potentially support HIV latency under cART, therefore this observation needs to be addressed equally, instead of bias their conclusion toward the CD127+ cells. Limitations should be addressed in regard to this findings and the in vitro data.*

In response to this comment, we have added to our results section, immediately after our description of the data that HIV DNA is detected in both populations, the following statement: “Although these results do not suggest preferential persistence of HIV in tissue-derived CD127+ Tm cells, they nonetheless demonstrate that these cells can serve as a cellular source of HIV persistence *in vivo*.” (Lines 231-233 in clean version).

**Reviewer #3**

Minor Issues

*Need to provide a better rationale for controls to normalize DNA levels in fig 3.*

In response to this suggestion, in the Results section, after we present the data that mitochondrial content was similar between all the populations of sorted cells, we now state that this method is a one that “has previously been implemented to normalize for levels of integrated HIV-1 provirus” (Lines 209-210 in clean version) and then cite the following two papers: Reinhard et al 2014, Retrovirology 11:12, and Butler et al 2001, Nat Med 7:5:631. This provides the rationale for normalizing DNA levels to mitochondrial content. Then, in the legend of Figure 3E, we have clarified that the data reported correspond to the HIV DNA levels normalized to mitochondrial content, divided by the productive infection rate as defined by the frequency of HSA-expressing cells. As stated in Lines 213-218 of the clean version, this way of reporting the data was used to demonstrate that the levels of integrated HIV DNA relative to productive infection rates was disproportionately high in the CD127+ Tm cells.

*Figure 3 labeling needs to be corrected*

We apologize for this labeling mistake, and thank the reviewer for catching this. Panel C of Figure 3 has now been corrected.
